# On Diseases Resembling Syphilis

**Published:** 1818-07-01

**Authors:** 

**Affiliations:** formerly House Surgeon to the Lock Hospital


### Medico-Chirurgical Journal;

OR,

### Quarterly Register

OF

### MEDICAL AND SURGICAL SCIENCE.

[ FIRST QUARTERLY SERIES. ]

No. 1.] JULY 1, 1818. [Vol. I.

### First Department.

### PRACTICAL ESSAYS AND COMMUNICATIONS, *Original or Translated.*

### I.

### *On DISEASES resembling SYPHILIS.*

By Dr. Burder, formerly House Surgeon to the Lock Hospital.

PRELIMINARY REMARKS BY THE EDITOR.

A More important question than that respecting the distinction between syphilitic and syphiloid diseases, has not agitated the medical world for many years. There never was a point of practice where Surgeons ran into such extremes as in the treatment of these diseases. A considerable portion of medical practitioners view the distinction above-mentioned as worse than useless; considering the syphiloid as merely modifications of the syphilitic affections, and requiring *mercury* for their certain eradication. Another class has lately started, to propagate the doctrine, or rather the practice, of curing syphilis and pseudo-syphilis, in all their Proteian forms, without any mercurial preparation; thus completely illustrating the line of the Poet,

Incidit in Scyllam cupiens vitare Charybdim.

There is yet a third class, who, adopting the wise precept,

"In medio tutissimus ibis,"

look upon the distinction (at least, as far as it regards *practice)* between syphilis and pseudo-syphilis as founded in just and accurate observation; and who, consequently, neither rashly prescribe mercury in *all* forms of genital defedations, nor withhold it in those forms of venereal diseases, which the experience of ages has stamped with the character of genuine syphilis.

But it may be asked, what can have given origin, and that so lately, to these clashing opinions and practices? We conceive that the origin must be attributed to some of the following circumstances: 1st. The total error of our predecessors in employing mercury for the cure of syphilis, when it might have been cured without that remedy. 2d. The total change of the disease within these few years. 3d. The inaccuracy of distinction which yet obtains between syphilis and pseudo-syphilis.

1. That our predecessors, and indeed ourselves, should, till within these eighteen months, have been entirely in the dark respecting the necessity of employing mercury in the cure of syphilis, is rather too much for sober credence; and yet this inference is pretty freely drawn of late. It is supported, apparently, by this fact:—"We now (say the Anti-mercurialists) see every primary symptom of syphilis disappear without mercury; a fact that could not be hitherto ascertained, *because* these primary symptoms were all treated by this remedy, to which the cure was falsely ascribed." This reasoning is specious, but perhaps not quite convincing. In the early part of the late war, a disgraceful regulation prevailed in the British Navy, viz. that of stopping a certain sum out of the pay of seamen who contracted the venereal disease; which sum was appropriated to the Surgeon. This measure had a most injurious effect; for the seamen not liking either to lose their pay, or to have the word "Venereal" appear against their names at the pay-table, concealed their complaints, or *quacked* themselves till their constitutions were often ruined. The consequences were, that the primary symptoms almost invariably disappeared without mercury, and that too under all the disadvantages of a sailor's duty and irregularity of living; but at subsequent periods, of various duration, secondary symptoms broke out with terrible violence, and our Naval hospitals were filled with the horrible victims of Syphilis treated *without mercury!* The evil, at length, became so glaring, that it was found necessary to change the system entirely; and then, when the Surgeons were applied to early, and treated their patients with mercury, secondary symptoms almost disappeared from our fleets and hospitals. This faithful portrait may not be without its use and applicability at the present moment; and may serve as a clue to unravel some of tbe problems which now occupy the minds of practitioners.

2. As to the total change in the nature of the syphilitic virus, within these few years, the supposition is rather improbable, and not sufficiently supported by facts. But that circumstances, which we cannot pretend to explain, have rendered the *true syphilitic sore* much less frequent, and consequently the syphiloid abrasions, *comparatively,* more common than heretofore, we are free to admit, since it accords with our own personal experience. But still we believe that the poison *exists;* and that, *as such,* it can only be cured by mercury.

3. Notwithstanding all that has been written on the diagnosis between syphilis and pseudo-syphilis, there is still great uncertainty on this point. Nothing but experience in one of those great establishments where the various shades of these Proteian diseases can be contrasted and compared, on a large scale, can bring any thing like conclusive evidence before the public. It is with this view that we here rescue from the mouldering shelves of Alma Mater a most valuable document, drawn from the first source of knowledge on the subject, and recorded without any bias or preconceived theory. It is a record of facts and faithful observations, which, we confidently predict, will not only be read with interest, but *remembered* with advantage by the private practitioner.

---

The male organs of generation are not exempt from the influence of the more general causes of disease, while they are particularly liable to be acted upon by temporary external stimulus, and disordered secretions from the female organs. The venereal disease has not been known in Europe more than three centuries; but diseases of the genitals have certainly been observed in the earliest ages of Medicine. Celsus, in particular, has accurately described some species of ulcers situated on the penis; and although he does not even hint at their causes, thinking probably that they rose spontaneously, yet it is likely that some of them were the offspring of sexual intercourse. The antiquity, however, of syphiloid affectious does not rest on conjecture alone; it is clear, from the writings of ancient Physicians, that ulcers and urethral discharges not only appeared spontaneously, but had often sprung from commerce with unhealthy females, long before the venereal disease was introduced into Europe. The learned Astruc quotes many authors of the thirteenth and fourteenth centuries, who accurately described various diseases of these parts, as excoriations—puriform secretions—phymosis—paraphymosis, gangrenous and phagedenic ulcers, &c. some of which are said to have been induced—"*propter coitum cum muliere fœtida.*"

Panic-struck by the unresisted ravages which syphilis first committed, the physician and the patient totally forgot those minor affections to which the sexual organs had been prone, and which were now indiscriminately referred to the *Nova Pestis.* To such an extravagant length was this idea carried, that a simple ulcer of the fauces, from whatever source oraginating, caused the unhappy sufferer to be deserted by his friends, and left to die, or recover by the efforts of nature, unaided—unpitied, and alone! The Physicians themselves were not more accurate in their diagnoses, when they discovered the mercurial specific. Absorbed in admiration of the wonderful effects of that divine medicine in syphilis, they inferred from a false species of logic, that every morbid symptom, removed by the specific, must have the same origin. A similar unfortunate error too generally prevails, even in the present time. Many there are, who require no further proofs of the nature of syphilitic disease than that it arose after sexual intercourse—wears any, however fallacious resemblance to the common forms of the disease—and disappears during the exhibition of mercury. A clearer knowledge, however, of the morbid qualities of other poisons, has convinced physicians that causes essentially different in their nature, may produce symptoms that are parallel in many points, but which tend to different ends by different means. Thus in Frambesia, a disease indigenous in Africa, from whence it was introduced to the West Indies, the eruption, followed by ulceration, and frequently attended with violent pains of of the limbs, has been known to spread by actual contact, and to bear a considerable resemblance to lues venerea. But experience proved that frambesia attacked the human frame but once, and at a certain period of life:—that in its first stages it is exasperated by mercury; and that it gradually exhausts itself, and eventually disappears with little or no medical aid.

The disease called "Sivvens," and which has been so long known in the South East parts of Scotland, affords a still more striking example of pseudo-syphilis. Here, as in frambœsia, a peculiar contagion is transmitted from person to person. It is likewise followed by ulcers, eruptions, pains of the limbs, &c. but seldom, if ever, can the powers of nature remove them. Mercury, as in syphilis, is the only specific; yet, however strong the resemblance may be, we are constrained to acknowledge that syphilis and sivvens arise from two distinct sources.

The description first published by Gilchrist, and the cases subsequently by authors, particularly Dr. Adams, shew us that the first appearance of this disease is an aphthous speck or cutaneous ulcer; or more correctly speaking, a grey crust of coagulated lymph, the membrane beneath being inflamed. The succeeding ulcers are remarkable for a florid surface, and thin edge. They spread rapidly; and have not the callous edge and base of the true syphlitic sore. In sivvens, too, buboes are rarely, if ever, to be seen. If then, diseases so distinct in certain *principal symptoms,* have yet others which are common to both; it is at least probable that many morbid states, though proceeding from a very remote cause, and requiring different modes of treatment, may put on a tolerably exact resemblance of syphilis. There can be little doubt that certain diseases which attacked the genital organs in the time of Celsus, are equally prevalent at present; and since to those must be added many diseases of the same parts arising from the incautious use or abuse of mercury, and an old syphilitic taint, it will be acknowledged, that a wide field for investigation and discrimination lies open before us.

The superior genius of John Hunter first turned the attention of physicians to syphiloid diseases. The extraordinary symptoms consequent on transplantation of teeth, and resembling those of syphilis, but often vanishing spontaneously, gave him reason to suspect that many other morbid effects considered syphilitic, were not so in reality, This opinion first taken up by Hunter, was pushed to a still greater extent by his sagacious commentator, Dr. Adams, and his learned and celebrated admirer Mr. Abernethy. Much useful instruction has also been derived from the indefatigable labours of the illustrious John Pearson, whose great talents and extensive means of information have given him a wonderful insight into this Porteian malady. Still later, Mr. Carmichael has enriched the common stock of science by much valuable and curious matter.

*Causes of Syphiloid Diseases.*—An interesting point of inquiry first presents itself; namely, whether these diseases arise from certain modifications of the syphilitic virus, or from other totally distinct poisons? Hunter believed that new morbific poisons were daily swelling the list:—this opinion we can neither confute nor confirm, without considerable difficulty. A late intelligent writer, [FERGUSON *Med. Chir. Trans. Vol.* iv. *Art.* 1.] is confident that true syphilis becomes milder in proportion as it is more widely disseminated; provided that dissemination be not checked by the exhibition of mercury. He endeavours in this manner to account for the venereal disease attacking the inhabitants of Portugal and Russia in so mild a form, that it seldom requires any other remedy than sarsaparilla, or decoction of the woods, for its cure. But this poison produces such virulent effects on strangers as defy the power of mercury itself. It is certainly reasonable to suppose, that a man long exposed to the action of any morbid poison may become less easily injured by that virus; but it is by no means so likely that the distinctive marks of a poison of this kind should so totally disappear as to be with difficulty cognizable. From the information I have been fortunate enough to acquire from army surgeons who have served in those countries; and from the description of symptoms by the above-mentioned ingenious author, I should conclude that the disease is rather syphiloid than syphilitic. Its tendency to sloughing and phagedenic ulceration; its spontaneous disappearance; and the frequent aggravation of its symptoms under even a cautious exhibition of mercury, draw, I think, pretty clear lines of distinction between it and syphilis.

Without doubt, climate, constitution, greater or less diffusion, and different modes of treatment, may make considerable variation in the disease; but we can hardly believe that these circumstances will altogether change its nature. Among ourselves, notwithstanding the manifold and Proteian forms of pseudo-syphilitic diseases that are daily remarked, it is quite clear that syphilis itself has not lost any ground. Its presence is, at this day, distinguished by the very same pathognomonic marks that are handed down to us by the oldest authors who have seen and recorded the disease. We still see the hollow, sloughing, cineritious surface — the hardened edge and base of the ulcer; and these marks have remained unchanged, since the poison was first introduced into Europe. The disease, moreover, is known to yield to mercury alone. It seems therefore probable, that syphilis itself still retains its pristine nature and form; and that the syphiloid diseases described in modern times, either arise from some new morbid poisons, or are the same that attacked the human race before syphilis was known; but which, for a long time, were not distinguished from the effects of the syphilitic virus. If, indeed, we could prove that new poisons are generated, still we must expect to see, again and again, those same forms of disease attack the organs of generation, that were described so many ages ago. We cannot prove that *these* have been annihilated. However this may be, we are greatly embarrassed by the questionable and multiform shapes which these diseases assume.

The syphilitic virus, although its power is great, does not injure all that are exposed to its influence. Several men have been known to have had sexual intercourse with one infected female, at very short intervals, and yet but a small proportion of them contracted the poison. As it is highly probable that most of them came in actual contact with the virus, it seems that a pre-existent capability of infection, or kind of diathesis, is necessary for its reception. The effects of the virus producing syphiloid ulcers are still less universal; and the nature of the virus itself more varied. It is well known that many unfortunate prostitutes, although unconscious of being diseased, have infected numbers who have had commerce with them, with syphiloid diseases. Nay, it is beyond a doubt, that even the *natural secretions* of females who have promiscuous intercourse with men, who pay no regard to cleanliness, and who destroy their health, strength, and life, by excessive drinking, become so acrid, that few can have commerce with them with impunity. Yet, in these very females, the syphiloid discharges and syphilitic virus are totally distinct; since the female that communicates the former disease may have no ulceration, whereas the syphilitic virus can only proceed from a syphilitic sore. The true venereal tint is known by a peculiar and constant train of marks, but the syphiloid poison produces different effects, according to the condition of the part, and the constitution of the patient. The above-mentioned source of syphiloid virus, in all probability, gives rise, at one time or other, to every form of pseudo-syphilitic diseases; the great variety of which may depend on the great variety in the nature of the cause; for when we see a syphiloid ulceration excited by the matter of a virulent gonorhœa, we find its nature less changeable than when excited by a less specific cause. A still greater contrast between the origins of syphilis and pseudo-syphilis is exhibited in the examples of men contracting the latter from healthy and decent females. Many unequivocal instances of syphiloid ulcers have occurred in men who had no intercourse with any but their wives, whose character and freedom from local disease, precluded all idea of syphilitic taint. It is very remarkable, that in almost every example, impaired digestion, and even a general bad state of health, had preceded the syphiloid symptoms. We are forced then to conclude, either that a secret and unobserved change had taken place in the natural secretions of the wife, or, which is much more likely, that nothing of the kind would have happened, had not an unsound state of health in the husband increased the morbid diathesis previously existing in his constitution, and rendered it susceptible of impression, even from the natural and otherwise innocuous secretions of the female organs.

We must also recollect, that many syphiloid diseases arise without preceding sexual intercourse, or at a very long interval afterwards; in short, spontaneously, and without any apparent cause. In such cases, it cannot be expected that the organs of generation should be more affected than any other part of the body, unless predisposed by some morbid change. Where syphiloid diseases exist, then, without any external cause, the latter must be sought in a deteriorated state of the whole body; and particularly in that state produced by repeated and long continued courses of mercury, by frequent attacks of syphilis, by intemperance in the use of ardent spirits, by neglect of cleanliness, or by two or more of these causes combined. This mal-habit of body was aptly termed by Mr. Pearson, CACHEXIA SYPHILOIDEA, which he conceives to be either idiopathic, or arising from the above-mentioned causes. With these observations in view, let us proceed to the examination of syphiloid causes as predisposing and occasional.

The predisponent are the most numerous, and are either topical or general. *Local predisposition* may arise from the application of acrid secretions, either of the sebaceous glands, the matter of gonorrhœa, of preceding ulceration, or, in short, any irritating cause which renders the part more susceptible of subsequent irritation. General or constitutional predisposition may spring from a variety of causes that otherwise produce a bad habit of body; but, particularly from long and debilitating courses of mercury.

This differs materially from what has been termed *the mercurial disease,* inasmuch as it does not directly follow the exhibition of that medicine, but usually at a remote period. It also differs in many other essential points. So powerful, too, is sometimes the syphiloid diathesis, that eruptions, ulcerations, and other phenomena resembling syphilis, arise without any apparent exciting cause.

The *exciting causes* we have already alluded to; viz. diseased secretions affecting the healthy, or healthy secretions affecting the predisposed; the matter of virulent gonorrhœa, especially if applied to excoriated surfaces, &c. These will be best elucidated by examples.

The first is from notes taken at Mr. Pearson's Lectures.

1. A man six months after marriage, and who had had no commerce with any other than his wife, became affected with a urethral discharge, which Mr. Pearson cured. In a fortnight afterwards, ulcers arose on the extremity of the prepuce, which were also healed. Soon after this, the inguinal glands swelled, but subsided on the application of evaporating lotions. Two months afterwards, he had superficial copper-coloured spots on various parts of the body; and these too disappeared under the use of the decoctum sarsæ compos. This man never was affected with syphilis, nor did he ever use any mercury.

2. The next example shall be abridged from the writings of that excellent surgeon, Mr. Abernethy. A man who was married to a perfectly healthy woman, and who positively affirmed that he had had no connection with any other woman, became afflicted with a urethral running very much resembling a venereal gonorrhœa. This discharge was soon afterwards attended with swelling and partial ulceration of the penis. These symptoms were followed in succession by a bubo, ulcerated fauces, and cutaneous eruption—all resembling syphilis. The physician that attended him relied on his assertions of innocence, and abstained from the exhibition of mercury. All these morbid phenomena spontaneously disappeared.

Since writing the above I received a letter from John Pearson, Esq. Surgeon of the Lock Hospital, in which he gives so clear and apt an instance of *syphiloid disease,* arising from the first species of exciting cause mentioned, that I cannot avoid giving the reader a history of the case. Mr. Pearson says, in the same letter, that no less than five cases of married women, in many respects resembling the following case, have been seen by him in half a year.

"A man had a chancre on the interior surface of the prepuce, for which he went through the proper course of mercury, and was perfectly cured. About six months after his recovery he married a woman, who, in the fourth or fifth month after her marriage, had a small ulcer on the internal surface of the labia pudendi; which, however, was healed by some mild ointment. About three months after the ulcer was healed, the woman's health growing bad, an ulcer of the tonsil soon shewed itself, and copper-coloured blotches, covering the whole surface of the body, speedily followed. There was also great uneasiness in the muscles and joints. Her medical attendants, supposing the disease to be syphilitic, sent her to London: Mr. Pearson pronounced the disease not syphilitic, and cured the woman by the Portuguese decoction. Towards the end of the cure, all symptoms having disappeared, he prescribed small doses of mur. hydr. corros. and her health has now been good for two years. The husband had never any local or general symptoms of the disorder after the course of mercury to the present day. This case suggests the following queries:—Is it not probable, that though no disease shewed itself in the husband, either the former attack of syphilis, or the course of mercury, or both together, wrought some change of the secretions of the part that had first been affected? And might we not suppose, if such a change had been wrought, that it brought on the syphiloid disease in the wife, her constitution being easily infected, owing to the impaired state of her health?"

In both the two first examples the females were totally unconscious of disease; yet as the symptoms first shewed themselves on the genital organs, and afterwards ran through the same course with syphilis, we must conclude that some morbid matter was absorbed *in coitu,* which (the predisposition being in existence) gave rise to the syphiloid disease.—Here, too, we may glance at the symptoms related by Mr. Hunter as arising from the transplantation of teeth. Most of the subjects from whom the teeth were taken, were in perfect health. Nevertheless, in about a month after the transplantation into the sound jaw, an ulceration spread round the tooth; and eruptions, ulcerated fauces, and acute pains in various parts were the consequence, all disappearing without the aid of mercury.

Examples of syphiloid diseases arising from the acrid secretions of prostitutes, might be multiplied to a great amount, from the writings of Abernethy and other modern authors; but I shall only select one instance from a little work not much known. "Several young men," says Mr. Whitsed, (*Practical Remarks on Diseases resembling Syphilis, Peterborough*, 1813), "who had connexion with the same woman, were attacked with buboes in the groins. The woman soon afterwards left the place, and affected several other young men in a neighbouring city, with similar symptoms. In these, on the exhibition of mercury, the glandular tumours increased, and most of them sloughed or suppurated; but when the mercury was discontinued, the local affections quickly disappeared, and no constitutional symptoms ensued."

Of the third exciting cause which I have mentioned, namely, the matter of virulent gonorrhœa, I have lately met with an example, which I shall here relate. A. B. nineteen years of age, caught a slight gonorrhœa, and also lacerated the frenum preputii, the first time he had connection with a woman. The laceration was nearly healed at the end of ten days, by common applications, when small doses of calomel were given daily for two or three weeks, and the gums were made moderately sore. The gonorrhœa too was soon checked by an astringent injection. Shortly after the mercury was left off, the patient was affected with cough, and the common symptoms of cynanche tonsillaris. Aphthous specks, and small ulcerations soon appeared on the left tonsil, and by uniting formed an ugly ulcer. This ulcer soon spread wider and deeper, and copper-coloured spots had just made their appearance on the abdomen, when I first saw him. The history of the case, and the appearance of the symptoms, proved to my mind that the case was not syphilitic; and I prescribed the internal and external use of the nitric acid. The patient being otherwise healthy, only gentle purgatives were employed. In three weeks the ulcer of the tonsil, though spreading in one place, was healed almost entirely round, and the blotches had totally disappeared. Some excoriations, occasioned by the contact of the former matter of gonorrhœa on the penis and scrotum were also removed by a solution of sulphate of zinc. At the end of the fourth week, the left tonsil was almost healed, when a small ulcer was discernable on the right tonsil. The decoctum sarsæ was now used liberally, and with advantage; but at this period, the patient having imprudently exposed himself to wet and cold, was soon affected with general swelling of the lymphatic glands of the penis and groin, accompanied by much inflammation, tumefaction, and ulceration of the fauces, and much constitutional irritation. Then speedily followed, pimples of a vivid red colour, collected into small clusters on various parts of the body, scaly eruptions of a dark colour round the margin of the scalp, and a thickening of the periosteum of one tibia appearing suddenly and without pain. At this unlucky juncture, the patient took two, and only two, of the mercurial pills, formerly mentioned, which evidently increased the inflammatory symptoms, and brought on, or at least renewed, the ptyalism. Bleeding, the warm bath, purging, and diaphoretics abated a little the febrile symptoms, when sarsaparilla, with occasional doses of hyoscyamus and opium, were again exhibited. The ulceration of the fauces, which, in one place, had spread from the left tonsil to the uvula, and was proceeding from the right to the larynx, by degrees put off its frightful appearance, shewed a cleaner surface, and lost the white unequal edges by which it was surrounded; the crop of pimples, too, vanished, and the thickening of the periosteum soon disappeared. The fauces at length healed, and the patient got well. Now, if symptoms like the preceding, and so closely imitating those of syphilis, shew themselves after gonorrhœa, and yet may be cured without the use of mercury; is it not probable that many, if nòt all the cases supposed to be syphilitic, and attributed to the absorption of gonorrhœal matter, are, in reality, syphiloid?

Some singular examples of syphiloid disease, from ulcerated mamillæ, have fallen under my own observation. The following case occurred in the Lock Hospital, London, while I was House Surgeon in that Institution. M. Young, a respectable female servant, was affected, at the time of her admission, with inflammation, swelling, and slight ulceration of the tonsils, a scaly eruption of a pale red colour over the whole body, swellings on the shin bones, and a small ulcer in the corner of the mouth. She attributed these symptoms to her having nursed her sister's infant, after he had sucked a nurse for ten weeks, who was suspected to have syphilis. Eruptions had shewn themselves on the child's body a few weeks before his death; and one week after his decease the ulcer appeared in the corner of the servant's mouth. Her fauces were affected about the same time; and a few weeks after this, the eruption broke out. These symptoms entirely disappeared in about a month, by the use of the warm bath, diaphoretics, and decoction of sarsaparilla.

### *Diagnosis of Syphilis and Pseudo-Syphilis.*

1.—A certain unpleasant sensation accompanies and follows the breaking out of a chancre; and this increases more or less through every stage of it, even during the administration of mercury. Syphiloid ulcers, on the contrary, have often arrived at a considerable size, before they have become troublesome. In a few instances, however, they became suddenly and violently painful, but not in general, without some preceding uneasiness. 2.—A chancre generally begins like a pustule or vesicle, which becomes a small whitish or grey ulcer. But syphiloid sores, though they are sometimes pustular at the commencement, generally put on the appearance of an excoriation, or trifling ulceration. 3.—A chancre generally proceeds slowly and regularly, preserving more or less exactly an oval, or circular shape, and spreading evenly on all sides. But a syphiloid ulcer is generally irregular in its progress; it either gives no trouble at all, or spreads rapidly. It often spreads in one place while it heals in another. Sometimes it shews a tendency to heal, and the next day to spread. 4.—A chancre has generally a foul, ash-coloured, hollow surface (excepting when seated in the common integuments), with a thin slough, or thick pus tenaciously adhering to the centre, which is without granulations. A syphiloid ulcer, on the other hand, seldom shews a surface so evenly hollowed out—it is more irregular, superficial; and granulations are often detected in some part of it. A chancre may be particularly distinguished by its hard base, which extends beyond the ulcer—is circumscribed, and often deep seated. But in the syphiloid ulcer, there is either no hardness at all; or it is only slight and diffused—not circumscribed. 6.—At a very early period in chancre, its projecting, round, and callous edges are distinguishable. In pseudo-syphilis the edges are generally thin, flat, and more or less ragged—sometimes, however, they are round and callous; but this is only observed when the ulcer becomes chronic and languid; disappearing by the use of stimulants. 7.—Lastly, a chancre on the body of the penis, and which is not yet influenced by mercury, sometimes wears a livid hue, which, in a few days, changes to dark brown. Nothing of this kind appears in syphiloid ulcers. The time, too, that elapses before the chancre appears after infection, varies less; for it seldom shews itself before the 4th, or after the 14th day. Whereas the syphiloid ulceration often appears in 20 or 30 hours after sexual intercourse—sometimes not for weeks.

By some of the foregoing diagnostic marks, we may, in general, distinguish chancre from syphiloid ulceration. But in no case should we trust to *local* examination, however minute We should scrutinize with the greatest care the mode of life, the peculiarity of constitution, and the state of the health of the patient. These will often guide and fix our judgment, which is liable to err without such an investigation.

*Primary syphiloid ulcers* are,—1. *Excoriations* of the glans penis, often produced by the matter of virulent gonorrhœa, and often followed in turn by urethral discharge, particularly where the patient has been affected with strictures, or has had violent means used for the cure of previous gonorrhœa. These excoriations are rarely accompanied by any material loss of substance; but frequently shew an irritable surface, and spread fast in irregular directions. Mr. Pearson first discovered that one half of the glans penis frequently became affected, while the other half remained free; the latter becoming diseased as the former healed. I have seen the whole interior surface of the prepuce and exterior surface of the glans penis covered with syphiloid ulcerations of this kind. These are frequently mistaken for chancres, but not with impunity: they too often spread, with violent inflammation, on the exhibition of mercury. Cooling, and gently astringent lotions are found to be best suited to these ulcers. The constitutional syphiloid symptoms, in these cases, are also aggravated by mercury.

2. *Preputial herpes,* generally preceded by a sense of heat and itching, appears like small vesicles collected into clusters, that attain their full growth by the end of the second or third day. They now break, and disclose small excoriations, with white surfaces, and edges only slightly elevated; and not hard, unless irritated by improper applications. In eight or ten days, they generally begin to heal under thin brown scabs. The learned Dr. Bateman has observed these vesicles, situated on the exterior part of the prepuce, dry up, without any ulceration, on the ninth or tenth day, and turn into scabs. Mr. Pearson has seen the same. It is not yet ascertained whether this species of herpes ever rises spontaneously, where there has been neither venereal taint nor the stimulus of mercury: it is apt to recur.

3. *Aphthous Ulcers*—not preceded by vesicles, nor surrounded by inflammation; very superficial; generally covered by a little adherent matter; often coalescing. These are easily cured by simple cooling applications, and do not give rise to constitutional symptoms. Ulcers somewat similar shew themselves sometimes after the use of mercury, but *these* are preceded by vesicles, nor do they observe the course of herpes above described, nor throw out scabs, when they are healing.

4. *Tubercles* of the prepuce, generally round, and of a reddish purple colour; surface considerably elevated, and about the size of a walnut. When they break, they leave a deep, foul ulcer with high edges. They heal slowly; but not so much so as *venereal* tubercles. They do not seem to produce secondary symptoms. Mr. Pearson has observed them principally in gouty subjects, and those whose constitutions had been much injured by long courses of mercury.

5. *Superficial Ulcers*—with slightly elevated edges; generally uneven; without hardness of the edges or base, but surrounded by a red circle. They usually begin by pimples, and proceed gradually. The surface, which is commonly whitish, sometimes red, shews no appearance of granulations; and when carefully examined, seem either to be on a level with the surrounding parts, or to project a very little. Their chronic state is generally distinguished by a thickened edge. They sometimes accompany inveterate buboes, but seldom, if ever, produce secondary symptoms. The cure must be varied according to the degree of the inflammation. Gently astringent lotions are often useful; and Mr. Carmichael recommends the application of brown oxide of mercury.

6. *Ulcers of the Surface*—without either induration or raised edges, chiefly taking place on the external prepuce, scrotum, labia pudendi, and on the breasts of women who had suckled or nursed infected children. These ulcers have generally a polished, smooth appearance, without granulations, covered by a viscid matter. Ultimately the surface becomes fungous, healing perhaps in the centre, while it spreads widely around. These ulcers often put on a healthy look, on the exhibition of mercury; but soon become stationary, shewing thick and hardened edges. Constitutional symptoms, such as ulcerated fauces, and crops of pimples on the skin, are often the consequence of these ulcers. When irritable, the mildest applications only are to be used: afterwards, lotions containing nitric acid, or nitrate of silver.

7. *The Phagedenic Ulcer*—This is of a bright red colour, without granulations, having thin everted, or inverted edges, very irritable, and generally attended by more or less inflammation. It is often known to attack those who have drank too freely of raw spirits after sexual intercourse. It spreads with a fearful rapidity, and is sometimes surrounded by a livid circle; it exudes a thin corrosive liquor. This ulcer frequently arises spontaneously in syphiloid cachexy resulting from the excessive use or abuse of mercury. I have seen it creep along the ala nasi, and at last eat off a great part of the nose. Secondary symptoms follow this form; but they have not yet been sufficiently characterized to hazard a specification. This ulcer requires to be treated with very mild remedies; as every stimulating application is dangerous. Lotions and opiate fomentations externally, with the free exhibition of opium, hyoscyamus, conium internally, are often necessary. When the inflammatory symptoms run high, local and general blood-letting is essential. Mercury is injurious.

8. *Sloughing Ulcer*—is at first a good deal of the phagedenic nature; but proceeds more rapidly, and more by sloughing than ulceration. The phagedenic and sloughing ulcers frequently alternate. In some instances, the secretions of the same female will produce sloughing ulcers in several men, in succession; but in general, the nature of the phagedenic ulcer depends on the constitutional idiosyncrasy and predisposition of the man himself. Celsus has accurately described both the sloughing and ulcerating forms. I have seen the whole penis; nay the scrotum and testicle destroyed by the sloughing ulcer. Much inflammation usually attends this dreadful disease. The secondary symptoms are generally characterised by pustules and papulæ. Venesection must sometimes be had recourse to, to relieve the great irritation of the system. Purgatives, refrigerants, diaphoretics, opiates, are particularly necessary. The external applications must be lenient. After the tone of the system has been sufficiently lowered by evacuations; bark, wine, and the diffusible stimuli are useful.

9. We have already observed that syphiloid diseases may arise from virulent gonorrhœa. Mr. Pearson has, for years, described certain anomalous symptoms that occasionally follow this urethral flux, and thinks that it gives rise to one variety of syphiloid cachexy. I myself have seen many cases of this description. Whether or not constitutional symptoms ever follow simple gonorrhœa, without excoriation or abrasion of surface, seems to admit of doubt. Carmichael appears to think that they possibly may. But since so very many who labour under gonorrhœa escape these symptoms, while the greater part of those who experience them, have had excoriations, abrasions, or warts, it seems probable that some of the latter are necessary for the production of the former.

10. *Syphiloid Buboes.*—There is scarcely any primary syphiloid ulcer that will not occasionally produce irritation of the neighbouring lymphatic glands. But the excoriations from virulent gonorrhœa—superficial ulcers both with and without projecting edges, phagedena whether of the sloughing or ulcerating kind, most frequently give rise to syphiloid buboes. The latter, however, sometimes arise without any previous primary ulcer; and, when this is the case, affections of the skin and fauces are apt to follow. It appears to me, either that simple irritation is transferred to the nearest gland, not being equal to produce ulceration of the part; or that the vessels of the part absorb the morbid matter, and then transmit it to the glands in the vicinity, the part itself undergoing no morbid alteration. This kind of blind absorption must be owned to be very rare in true syphilis, the poison of which, generally, if not always, ulcerates the part it touches. When an ulcer does not precede a syphiloid bubo, much functional disturbance in the constitution usually ensues. In scrofulous subjects the abscess, for the most part, proceeds slowly, and turns to a troublesome suppuration without much pain, changing to an irregular, languid, and chronic ulcer. In other instances, however, the suppuration is rapid, and accompanied with much pain. The subsequent ulcer does not shew the thick edge and unequal surface of the former species, but is rather distinguished by thin edges, either inverted or everted; and a raw, red, angry surface. These ulcerations spread rapidly, attended with great constitutional derangement. It is much more difficult to distinguish between the diseases of the glands, than between the primary ulcers that arise on the genitals. I may, however, remark, that the syphiloid virus generally affects the *upper* row of glands in the groin; and when they break, they have not the copper-coloured edges, so often seen in the syphilitic bubo; nor do they proceed so regularly. The syphilitic virus, almost always, affects one, and only one gland of the middle row. The phagedenic bubo can hardly be taken for any other except that which arises from mercurial irritation. As for the languid syphiloid bubo of scrofulous habits, it has a totally different nature and course from that arising from syphilitic virus. The syphiloid bubo also spreads very irregularly. I have seen it spread upwards to the abdomen, and in other directions, while the original scite was quite healed. The treatment must vary with the symptoms; for a languid syphiloid bubo requires stimulating medicines; whereas a phagedenic or irritable one demands mild and lenient applications.

*Secondary Syphiloid Symptoms.*—These, the limits of this essay will not permit me to describe but with much brevity. As these frequently arise without any ulcer or local affection, we cannot deny them the term *constitutional.* The first decided mark of syphiloid cachexy, where no local affection preceded, is often an ulceration of the fauces; but, in general, it will be found on inqury, that the patient's health had not been good for some time previously to this affection. The ulceration of the fauces also is usually the first constitutional symptom that succeeds syphiloid bubo; but it seldom remains long without cutaneous eruption. This is not, however, the invariable succession of events. Flying pains are the frequent forerunners of faucial ulceration; and the cutaneous eruption is often preceded by general inflammatory symptoms.—The species to which belong constitutional *syphiloid* symptoms, is not so easily distinguished as are primary syphiloid sores, in consequence of the almost endless variety of the former: yet a careful inquiry into the history, and an accurate examination of the progressive symptoms, will generally lead to a true judgment of the case.

The affections of the fauces and of the skin are usually preceded by a sense of languor, restlessness, and inactivity. Every kind of attention is irksome; the spirits are depressed; the sleep is perturbed; the appetite fails; the tongue is whitish, and often coated with a viscid mucus; the bowels are torpid and irregular; the action of the heart and arteries is generally increased, the pulse being quick, sharp, and full. The skin, especially in the evenings, is unusually hot—all these symptoms having a remission towards the morning, with universal perspiration. The pains in the head are sometimes excessive and obstinate, while those in other parts of the body, at first fugitive, become more stationary in the arms and legs, being often mistaken for rheumatism. In a few days the patient is unexpectedly annoyed by a cutaneous eruption, or by some difficulty in swallowing; which, when we examine the throat, we find to depend on an ulceration of the *velum pendulum palati*, or of one of the *tonsils*. When these phenomena are fully established, there is generally a slight remission of the febrile irritation; but, if the disease prove obstinate, the fever returns with increased violence, and with more of the hectic appearance.

In syphiloid diseases, it is well worthy of remark, that constitutional symptoms, as ulcerated fauces and eruptions, frequently appear within the second, third, and fourth week after infection; so that we have seen in the same person, and at one and the same time, a primary ulcer of the genitals, and affections of the throat, skin, and periosteum. This wonderfully rapid progress, and simultaneous existence of two different sets of symptoms, will form a strong line of demarcation between syphiloid and syphilitic virus; for in the latter, secondary symptoms seldom shew themselves at so early a period, and affections of the membranes and bones *follow* those of the fauces and skin (unless resisted by mercury), as regularly as the latter follow a primary bubo or chancre.

*Syphiloid Affections of the Fauces.*—These are so dependent on, and modified by the general state of health, that it is difficult to give any description that may comprise the different varieties. I shall only trace three of these, which fell under my own observation.

1. *Chronic Ulcer with raised Edges.*—This, in general, first appears on the tonsils; is accompanied with very little pain, and liable to be mistaken for a syphilitic ulcer. It has, however, a more uneven surface than the syphilitic sore; its edges are less round and callous; the loss of substance less apparent; it is not covered by that gelatinous, whitish, or grey secretion; or if it is, it is easily washed off. Granulations may generally be discerned in some part of the ulcer; which, like the primary sore, seems to spread irregularly, healing in one place and extending in another.

2. *The Phagedenic Ulcer* is sufficiently distinct from the syphilitic, because it has neither thick, white, nor rounded edges, but a thin transparent margin. The surface too, which is more or less red and dry, is very irritable, with a disposition to slough. It is, in general, excessively painful, spreads very fast, and frequently attacks the posterior fauces.

3. *Superficial Ulceration of the Fauces.*—This extends more like an excoriation than an ulcer, and is usually accompanied by an erysipelatous inflammation of the fauces. In this affection, the vela pendula palati sometimes become perforated. The complaint is easily distinguished from venereal ulceration.

*Syphiloid Eruptions.*—The varieties of these are by no means, as yet, well defined. The most common syphiloid eruption is known by *papulæ,* of a clear red or copper-colour, gathering into irregular clusters, and slightly raised above the surface. Sometimes these papulæ, as they proceed, put on the appearance of vesicles or pustules. In general, however, they preserve the *papular* character until they undergo desquamation. In some instances, the syphiloid eruption more nearly resembles the common plain syphilitic blotch; because very suddenly, and before any remedy has been applied, it becomes squamous. Of this nature was the eruption of Margaret Young, already noticed. Abernethy has faithfully delineated this eruption, which shews itself in many cases of pseudo-syphilis. We may distinguish the *squamous syphiloid blotch* from the *copper-coloured syphilitic eruption* by the following marks. The *former* is of a brighter colour, feels rough to the touch, and is less depressed in the centre. On close examination, it will be found to be *many-sided,* and not a smooth *circular* area, as in the syphilitic blotch. It leaves behind it no mark or depression of the skin.

Dr. Bateman has lately described a *tubercular* eruption of the syphiloid kind, attended by ulceration of the fauces, but without previous affection of the genitals. One woman thought she had caught the infection from her husband, who, two months before, had laboured under a similar disease. The tubercles, of a reddish colour, smooth, and shining, are one or two lines in diameter, rise barely above the surface, and have a depression on the top. The disorder generally disappears in a fortnight or three weeks under a course of bark, mineral acids, and sarsaparilla.

The *papular* eruption, as before observed, more frequently than any other, follows primary syphiloid ulcers. A *phagedenic* ulcer, however, is often followed by pustules with inflamed bases, and apt to terminate in ulcers with fungous excrescences. It is probable that syphiloid eruptions appear under as great a variety of forms, as those cutaneous defædations that arise from syphilitic virus. Dr. Willan has noticed several of *these.* Pearson also, among others, has described a pustular eruption very much resembling variola, and leaving pits in the skin; an eruption of tubercles of an obscure red colour without pus, and the common squamous maculæ. These three he considers as syphilitic. I have myself seen eruptions both of pustules and papulæ, which, from concomitant symptoms of lues, and their giving way to mercury, I concluded to be truly syphilitic.

With these demonstrative proofs in view, we cannot but wonder at the opinion of a late learned author, Mr. Carmichael, who asserts that the squamous blotch is peculiar to syphilis, and never seen in pseudo-syphilis; whereas, although eruptions of papulæ and pustules are often seen in the *latter,* they are never found in true syphilis.

### *Syphiloid Affections of the Membranes and Bones.*

The pains in pseudo-syphilis are generally excessive, especially towards night; and yet the nocturnal exacerbation is not so distinctly marked as in real syphilis. The syphiloid pains are less confined to certain parts than the syphilitic; they often change their situation, and do not attack the middle only of the cylindrical bones, as in the other disease; but the great joints also, so as to be often mistaken for rheumatism. A frequent symptom of pseudo-syphilis is a chronic inflammation of the synovial membranes of these joints, not unfrequently accompanied by a considerable effusion into their cavities. The bones themselves, as far as my observations extend, are seldom affected early in syphiloid diseases, except where the complaint is exasperated by the use of mercury. The periosteum is first affected by inflammation, and effusion beneath it and between its tissues. Induration and thickening are the consequences. This kind of node is of an irregular appearance, and rarely gives much pain. Sometimes they appear very suddenly on the head, the clavicles, the ulnæ, and the tibiæ. I have, myself, seen them rise and vanish in the space of seventy hours. In these instances, the tumours were soft and compressible; apparently effused serum or coagulable lymph.

### *Treatment.*

When we survey the various causes, predisposing and exciting, of syphiloid diseases, it may readily be supposed that it is a very difficult task to lay down explicit and simple indications of cure in all cases. We have seen that these diseases may arise spontaneously, or, more properly speaking, without any exciting cause; and we have also seen that they may come on as the remote effects or consequences of a long or injudicious course of mercury. Their most frequent origin, however, may be traced to the absorption of some irritating matter. In syphiloid diseases, it is necessary to remember, that, contrary to what takes place in syphilis, the symptoms gradually exhaust themselves, as it were; and, unless exasperated by injudicious treatment, disappear without medical aid. In this respect many syphiloid diseases intimately resemble frambesia, which, though a chronic and tedious complaint, runs its stated period, and vanishes spontaneously. The *local* treatment of syphiloid affections has already been glanced at, but it must depend on the general principles of surgery, the state of the health being particularly attended to. The three following indications may probably include the principal remedial measures which it is desirable to pursue. 1. To weaken as much as possible the poison disseminated in the body. 2. To oppose and counteract its effects. 3. To support the strength of the patient.

It is to be regretted, that no specific has yet been discovered that is capable of effecting the first indication. Mercury, which is so wonderfully efficacious in subduing the syphilitic virus, seems rather to irritate than assuage the acute stages of syphiloid diseases. We must therefore beware of exhibiting this mineral when there is much ulceration, and particularly where there is any phagedenic or irritable ulcer, which is always exasperated by a stimulus of this kind. Mercury, however, when carefully and sparingly exhibited, is often observed to heal speedily the chronic species of syphiloid ulcers. If the doses be moderate, and the course not too long persisted in, this favourable change may be permanent. But too often, the practitioner, elate with success, is imprudently led to push the medicine to the full effect of ptyalism, when dreadful symptoms, in general, ensue, and the disease continues to harrass the patient for months, or even years. We may, therefore, employ mercury to alter to a more healthy condition, those parts affected by languid and chronic pseudo-syphilis; taking care to use the mildest preparations, as the blue pill, which must be very soon left off, lest mercurial iritation attack the constitution. For as, on one hand, these diseases are known to exhaust themselves; so, on the other, mercury, however useful it may be for a time, is found, if long continued, to prove injurious to the healing powers of the constitution, and finally to render the disease more inveterate and incurable.

Sarsaparilla, prepared according to the *Ed. Pharm.* and administered in large doses with mild antimonial diaphoretics, is possessed of no mean efficacy in syphiloid diseases, whether arising spontaneously, or from evident causes, especially in weak habits, and after the abuse of mercury. I have so repeatedly seen ulcers of the fauces and other parts, pains, and obstinate cutaneous defædations yield to this remedy, that I cannot speak too highly in its praise, especially as relapses after its use were rare. But there is no security in its exhibition unless the dose be from one to two pints daily.

To answer the second indication; namely, to resist the effects of the poison, we must direct our attention to the most urgent symptoms. Thus, during the time that an ulcer is rapidly spreading, and sometimes while eruptions on the skin are coming out, great irritability of the system prevails, and requires our serious notice. In robust constitutions, and where there is much increase of vascular action, venesection will, in general, be useful, and in most cases, purgatives, diaphoretics, with or without opium, diluents, the warm bath, and low diet, will moderate the local and constitutional symptoms, and prepare the system for sarsaparilla, the mineral acids, and other tonic or restorative remedies.

The irritation and pain are sometimes so urgent, that full and repeated doses of opium, henbane, conium, lactuca virosa, &c. are necessary; and ought to be administered, in these instances, until some remission of the pain or manifest effect ensue. In three very dangerous cases of syphiloid ulceration which resisted every other means, large doses of opium suddenly and wonderfully mitigated the symptoms, as soon as the pain and irritation were soothed. The doses were then gradually and cautiously diminished. The greatest attention must be paid, in all syphiloid diseases, to the state of the chylopoietic organs, which exert a surprising influence over both local and constitutional affections.

The last indication, which regards the support of strength, is often one of no slight importance. In syphiloid cachexy it too frequently happens that we can do little more than palliate the more urgent symptoms, and keep up the strength, while the disease runs its course, or is overcome by the powers of the constitution. The latter therefore must be assisted by nourishing, but not stimulating diet, by gentle exercise, good air, and the moderate use of tonics. The warm sea-bath is often of singular service. Sarsaparilla has been already mentioned. Bark, and the various preparations of iron are sometimes useful; but, in general, they do not produce such certain effects in syphiloid diseases, as in some other kinds of debility. Fowler's solution of arsenic I have sometimes known to give temporary relief. The milder preparations of mercury, in very small doses, are often beneficial in the latter stages of the disease, the same as in frambesia. Its exhibition, however, as I remarked before, ought always to be limited to a short period, and the least approach to ptyalism or mercurial irritation should be carefully guarded against by leaving off the medicine.

The following case, which the kindness of Dr. Munroe, jun. Professor of Anatomy and Surgery in the University of Edinburgh, has favoured me with, clearly proves that superficial diseases of the bones sometimes arise spontaneously. These affections in adults are too frequently attributed to syphilis: mercury too, will sometimes give a temporary relief to these affections of the bones and their circumvesting membranes, while it increases the morbid predisposition to a future attack.

"A boy, aged eight years, who had previously enjoyed good health, was attacked without evident cause, with acute pain in the right side of the forehead, which was succeeded by a swelling of the upper jaw of that side. The swelling gradually increased until the eye-ball and the whole superior maxillary bone were considerably protruded. Although there was no suspicion of a syphilitic taint, mercury was prescribed. Under its action, the bone detumefied, and the eye-ball returned into its natural situation. After the medicine, however, had been continued about eight weeks, a dark red spot appeared on the middle of the *palatum molle,* which was succeeded by extensive ulceration of that part, as well as caries of the palatine process of the superior maxillary bone, leaving an opening as large as a shilling. The vomer was at length also destroyed. At this period, country air and a course of cicuta completely arrested the course of the disease, and health was ultimately restored."

### Remarks by the Editor.

If such an establishment as the *Lock Hospital* can be considered capable of affording strong evidence on a *venereal* question, we appeal to our professional brethren, whether the foregoing authentic document does not bear us out in our preliminary observations, and prove that, for all practical purposes, there are sufficient grounds for a distinction between syphilitic and syphiloid diseases, the *former* requiring the administration of mercury equally as much as at any former period. If this be the case, an accurate *diagnosis* of the two diseases is one of the greatest desiderata of the present day, and we trust that the foregoing article will be found a valuable contribution to this desired object.

